# Association between Functional Movements Skills and Health Indicators in Children Aged between 9 and 12 Years Old

**DOI:** 10.3390/ijerph14091010

**Published:** 2017-09-04

**Authors:** Megan E. Comeau, Danielle R. Bouchard, Cindy Levesque, Michel J. Jonhson, Brittany V. Rioux, Andrea Mayo, Martin Sénéchal

**Affiliations:** 1 Cardiometabolic Exercise & Lifestyle Lab, University of New Brunswick, Fredericton, NB E3B 5A3, Canada; Megan.Comeau@unb.ca (M.E.C.); dboucha1@unb.ca (D.R.B.); rioux.brittany@unb.ca (B.V.R.); Andrea.Mayo@unb.ca (A.M); 2 Faculty of Kinesiology, University of New Brunswick, Fredericton, NB E3B 5A3, Canada; 3 Sports New Brunswick, Fredericton, NB E3B 6A2, Canada; nbphysicalliteracy@sportnb.com; 4 École de Kinésiologie et de Loisir, Université de Moncton, Moncton, NB E1A 3E9, Canada; michel.johnson@umoncton.ca; 5 Faculté des Sciences de la Santé et des Services Communautaires, Université de Moncton, Moncton, NB E1A 3E9, Canada

**Keywords:** physical literacy, inactivity, waist circumference, physical active

## Abstract

*Background*: Children’s health is a current concern and data suggests that poor fundamental movement skills (FMS) could be associated with poor health, which may or may not be mediated by low physical activity level. However, tools to assess FMS have not been standardized, and could consequently lead to different associations between FMS and health indicators. *Objective*: The primary objective of this study was to evaluate the associations between FMS and health indicators using two different FMS measurement tools often used in Canada. *Methods*: A total of 145 children between the ages of 9 to 12 were recruited from schools, after school programs, and summer camps in 2016. FMS were evaluated using the Passport for Life (bound, plank, run, kick, throw) and the PLAYbasic (run, hop, throw, kick, and balance). The association between each test and an average score for each tool were tests with health indicators including anthropometric measures, grip strength, cardiorespiratory fitness, and percent body fat. *Results*: Participants were composed of 54.2% boys aged 10.4 ± 1.2 years with an average body mass index of 18.8 ± 3.8 kg/m^2^. The association between the average score of both tools was 0.77 (*p* < 0.01), body mass index was significantly associated with 67% of FMS elements using the Passport for Life (*r* ranging from −0.18 to −0.32; *p* < 0.05), and 60% of FMS using the PLAYbasic (*r* ranging from −0.15 to −0.30; *p* < 0.05). There were no significant differences between the associations of the health indicators with FMS and either FMS assessment tool (Passport for Life and PLAYbasic) (*p* = 0.05). Average score of FMS was significantly associated with all health indicators using both PLAYbasic and Passport for Life (all *p* < 0.05). *Conclusions*: Health indicators in children are associated with FMS regardless of whether the Passport for Life or the PLAYbasic was used as the assessment tool. It is worth investigating if interventions that improve FMS lead to improvements in these health indicators.

## 1. Introduction

Data from the Canadian Health Measures Survey 2007–2009 revealed that the overall health and fitness levels of children have significantly declined compared to children in 1981 [1]. Some data supports that fundamental movement skills (FMS) are associated with physical activity levels [2]. Failure to develop these skills during childhood might impact children’s physical activity levels in adulthood [3]. For example, a study found that physical activity participation was increased by 21% after 2-year follow-up in children with the highest baseline FMS in 293 children [4]. Interestingly, these observations might be preserved until adulthood, as a study that investigated the impact of FMS at 6 years of age on physical activity levels found that children with poor FMS had poor physical activity levels at 26 years of age [3]. Similarly, a prospective study of 330 children suggested that FMS was also associated with moderate-to-vigorous physical activity at 6-year follow up [5]. Many studies have focused on the relationship between FMS and physical activity, while very little investigated FMS and health indicators [1,6]. Altogether, these findings are of great concern as most children are not sufficiently active and the common belief is that most of the health indicators benefits occur through physical activity [1,6].

Health indicators, such as body weight and body mass index (BMI), have been associated with FMS [7,8,9,10,11,12]. For example, a study led by Vameghi et al. [7]. found that BMI variables had a significant effect on jumping, skipping, hopping and ladder climbing. In addition, poor FMS was negatively associated with obesity status in children aged between four and six [11]. and even younger (e.g., aged between 2 and 4) [13]. However, in terms of health indicators other than those related to body weight, not much information is available, and tools to assess FMS have not been standardized. Therefore, this could consequently lead to different associations between FMS and health indicators.

Tools to assess FMS vary and are complicated to validate because of the absence of a gold standard measure. As of now, in Canada, as an example, two tools have been gaining popularity to assess FMS: PLAYbasic [14]. and Passport for Life [15]. The PLAYbasic tool was developed in close collaboration with the Public Health Agency of Canada while the Passport for Life tool was initiated by Physical & Health Education Canada. Even if both tools assess FMS, they use different movements, evaluate a different number of actions, and calculate scores differently. To our knowledge, only one study has investigated whether children who are evaluated using different tools have the same associations with the studied outcomes [16].

The main objective of this study was to evaluate the association between FMS and health indicators defined as body weight, BMI, waist circumference, percent body fat, handgrip strength, and cardiorespiratory fitness using the PLAYbasic and Passport for Life tools. The second objective of this study was to evaluate whether the associations between FMS and health indicators were different between the two tools. We hypothesized an association between FMS and health indicators. Our second hypothesis was that both PLAYbasic and Passport for Life will display similar associations between FMS and health indicators.

## 2. Methods

### 2.1. Study Design and Population 

This study was conducted through a partnership between our research group, Sports NB, and the Royal Bank of Canada. The study consisted of 145 participants who were recruited through various schools, after-school programs, and summer camps across New Brunswick between April and August 2016. The participants were aged between 9 and 12 years old (grades 4–6) and both the children and their parents provided written consent before participation. This research was approved by the Research Ethics Board of the University of New Brunswick (REB:2016-047). 

The primary exposure variable was fundamental movement skills (FMS) measured using two widespread tools in Canada: PLAYbasic [14] and Passport for Life [15]. Each assessor received an accredited training on FMS. This training was performed through the Canadian Sports National Center. Inter-individual tests were performed to eliminate the subjectivity of assessors. Every evaluator received the Fundamental Movement Skills Workshop from the National Coaching Certification Program after which the inter-reliability was assessed with the person delivering the certification. After the 1-day training evaluators were tested against the expert’s scores. A coefficient of 0.9 between the expert and assessor was needed to be an evaluator for the study.

### 2.2. PLAYbasic 

PLAYbasic is a tool developed to evaluate FMS using five different tasks: run there and back, hop, overhand throw, kick ball, and balance walk backward [17] (described below). For each test, the assessor uses a 100mm scale with sub-categories of Initial (0–25), Emerging (25–50), Competent (50–75), and Proficient (75–100). This allows the assessor to be more specific with identifying the child’s ability for each task. Once the evaluator had decided the sub-category, the exact score was based on the strict criteria for each movement. For example, for the hop, Initial includes three criteria: Maintain single leg support, performs a jumping action, and synchrony between upper body and lower body movements. If a child is presenting one out of these three criteria, he or she would receive a score of 8.33 on a total of 25 points. All the details for each sub-category for all tests are available in the assessor’s manual [17]. After, the tests are averaged to have a sense of FMS competency: 

*Run There and Back:* The child must run from one cone to another cone, which are five meters apart, as quickly as they can. When they reach the second cone, the child must control their body to a complete stop, and run back to the first cone as fast as they can. 

*Hop:* The child stands next to a cone on one foot. They must hop laterally to a second cone that is one meter away, and land on their opposite foot. Finally, they must return to the original cone and balance on their original foot and stop without losing balance.

*Overhand Throw*: The child stands behind a line three meters away from the wall. They must throw the ball to a target on the wall that is 1.5 m above the ground. Then, when the ball returns, they must catch it with the opposite hand. 

*Kick Ball:* The child stands behind a line that is four meters from the wall. They must kick a stationary ball towards the target on the wall, which is one meter above the ground. The child must then perform a drop kick. With the same dimensions as the first kick, the child must start by holding the ball in their hands, release the ball, and kick it towards the target before it hits the ground.

*Balance Walk Backwards:* The child is instructed to walk backward “toe-to-heel” as quickly as they can from one pylon to another placed two meters apart on the floor while keeping their balance.

### 2.3. Passport for Life

Passport for Life is a FMS assessment tool that includes six different tasks: lateral bound, plank, run, throw and catch, kick, and a circuit that includes different tasks. For each test, the child earns a score from 1 to 4. These scores represent whether the skills were (1) emerging, (2) developing, (3) acquired, or (4) accomplished, based on strict elements identified in each movement as specified by the Passport for Life. Based on these elements, the average score of the tests are determined by the assessor in order to provide a sense of FMS competency. All the assessors instructed children on the task to perform without any additional details or cues, and therefore the actual movement was assessed. The assessment details can be found online [15].

*Lateral Bound:* The child starts by standing on one line, balancing on only their right leg. Then, the child must bound to another line that is 75 cm from the first line and land on their left foot. Without pausing, the child bounds back to the first line to land on their right foot. The child performs three trials and the best score is recorded.

*Plank:* The child begins in the table top position—Elbows resting on the floor underneath their shoulders and their knees on the ground underneath their hips. Then, the child must slowly bring their legs back one at a time until it is only the child’s feet, elbows, and forearms which remain on the ground. The child must maintain this position for a maximum of 60 s. If the child breaks the plank before the 60 s, another trial of 60 s is completed.

*Run, Stop, Return*: The child begins at the starting line, and runs as fast as they can to a cone placed seven meters away. The child then controls themselves to a stop, and runs back to the starting line. The child is allowed 30 s of rest before they complete a second trial. 

*Throw and Catch:* The child stands behind a line that is one meter away from a wall. The child must throw the ball so that it bounces on the ground before it reaches the wall. The ball should then hit the wall at least one meter above the ground. Finally, the child must catch the ball as it returns with one hand before the ball hits the ground. They must catch the ball only once it crosses the line that is one meter from the wall. The child completes one practice trail, which is followed by two recorded trials. 

*Kicks:* The first kick the child must complete is the place kick. The child stands behind a line that is four meters away from the wall. The child is aiming to hit above the target on the wall that is one meter above the ground. If the child is successful during the first trial, they progress to the punt kick. A successful kick refers on one in which the child steps forward with the opposite foot, makes solid contact with the ball (no stumbling or tripping), and kicks the ball over the line. If the child kicks the ball over the line on the first trial, they move on to the punt kick. The child begins this task holding the ball in their hand. Then, the child releases the ball from their hand and kicks the ball towards the target before it touches the ground. 

*Circuit*: The circuit is a four-station circuit that was designed to assess the ability of the child to participate in vigorous physical activity and consequently estimate cardiorespiratory fitness. The first station is an “Agility Ladder” where the child moves in a hopscotch pattern (e.g., two-foot hop in first square, two feet straddling the ladder, two-foot hop into the second square) up and then back through the ladder. The second station is “Ball Jumps” where the child starts in a squat position to pick up the ball and jump, raising the ball over their head. When the child lands, they squat down to touch the floor with the ball and then jump up again. The third station is “Figure Eights” where child stands between two cones facing one side/alley of a badminton court. The child’s shoulders and hips must face the same side of the field/gymnasium. The child moves their feet forward and then backwards to take them in a figure-eight pattern around the cones. The fourth station is “Scissors” where the child places one foot on each side of a line on the ground and switches their feet back and forth continuously. Each station is performed for 30 s each for a maximum of 12 min. The tested child must run between stations and are instructed to do so. Assessors record whether the participant walks or pauses between the stations. 

### 2.4. Primary Outcomes

The primary outcomes were anthropometric measures, muscular handgrip strength, cardiorespiratory fitness, and body fat percentage: 

*Anthropometric Measures:* Body weight was measured to the nearest 0.1 kg on a calibrated balance (BC-533 InnerScan Body Composition Monitor, Tanita, Arlington Heights, IL, USA) and height was obtained with a standard stadiometer (SECA gmbh & co. kg model #213, Hamburg, Germany). Body mass index (BMI) was then calculated using the following formula: body weight (kg)/height (m^2^). The BMI z-score was calculated using the CDC reference chart. The protocol was based on the participants age at the time the measurements were taken. Waist circumference was measured with a measuring tape to the nearest 0.5 cm between the iliac crest and the floating rib following a normal expiration. Participants placed their arms across their chest with arms on their shoulders according to protocol from the Canadian Society of Exercise Physiology (CSEP) [18].

*Grip Strength:* To complete this task the CSEP protocol was followed [18]. Briefly, the child stood in an upright position and held a dynamometer (Jamar Hydraulic Hand Dynamometer, Patterson Medical, Nottinghamshire, UK) in one hand. The dynamometer was in line with the child’s forearm and at 45 degrees angle. The child took a deep breath in and upon exhalation, the child then firmly squeezed the dynamometer until they reached their maximum capacity. The highest score from each side was added together for a total score and was used in the analysis.

*Cardiorespiratory Fitness:* Cardiorespiratory fitness was assessed using the shuttle test [19]. The appraiser first demonstrated the PACER Shuttle Run to the children by completing a few runs. The children completed a series of runs (with incrementing speed) between two lines that were 20 m apart. At the first beep, the child ran from one line to the other where they waited for a second beep. At the second beep, the child ran back to the first line. With each minute, the beeps got closer together and the child had to run faster to succeed. The child must have reached the line before they heard a beep. If the child didn’t cross the line in time, they received one warning. When a child didn’t cross the line in time for a second time, they were eliminated. Children were eliminated discretely: the appraisers simply recorded their elimination without telling the child they have been eliminated. Cardiorespiratory fitness was estimated using equations developed by Leger in 1982 [19]. VO_2peak_ was reported scale to kg of body weight. Because of the setting of the study, a sub-sample performed this assessment (*n* = 42).

*Body Fat Percentage:* A bioelectric impedance scale (Tanita BC-568 Inner-Scan Segmental Body Composition Monitor, Tanita, Arligton, IL, USA) was used to estimate body fat percentage [20]. To complete this assessment, each child stood on the bioelectrical impendence platform with a grip in each hand. From this position, a small electrical current was passed through the child’s body and resistance to this electrical current was measured, from which water percentage was estimated [20]. From this estimation, body fat percentage can be estimated. 

### 2.5. Statistical Analysis

Continuous variables are presented as means ± standard deviations, while categorical variables are presented as *n* (%). An average composite score was created for both PLAYbasic and Passport for LIFE according to the following formula: PLAYbasic composite score = [*Run There and Back (score) + hop (score)* + *Overhand Throw (score) + kick ball (score) + Balance Walk Backwards (score)*]*/5;* and Passport for LIFE composite score: [*Lateral Bound (score) + Plank (score) + Run, Stop, Return (score)* + *Throw and Catch (score) + kicks (score) + circuit (score)*]*/6.* Pearson’s correlational tests were performed to test the relationship between FMS and health indicators. Multiple linear regressions adjusted for confounders were performed to investigate the independent associations between FMS and health indicators. Linear regressions were conducted to quantify the magnitude of the variance explaining health indicators. Finally, to test the differences between the correlations given from the two FMS tools, and each health indicator, Fisher r-to-z transformations were used after getting the average correlation for each heath indicator. 

## 3. Results

### 3.1. General Characteristics of the Sample

The average age of participants was 10.4 ± 1.2 years (Table 1). The study was comprised of 54.2% boys. The average body weight of the participants was 39.8 ± 9.3 kg and the average BMI was 18.5 ± 2.9 kg/m^2^. The participants had an average waist circumference of 69.0 ± 8.7 cm. The average handgrip strength was 74.8 ± 21.9 lbs, while average cardiorespiratory fitness was 48.1 ± 3.9 mL kg^−1^ min^−1^. 

For the FMS measures, participants had an average score of 63.0 ± 19.0 for the PLAYbasic (out of 100) and 2.8 ± 0.7 (out of 4) for the Passport for Life. The association between the average scores of the two tools was 0.77 (p < 0.01). For the two tasks that are the same from one tool to the other, kicking and running, the correlations were 0.72 and 0.82 respectively (p < 0.01).

### 3.2. Association Between Health Indicators and FMS 

Using PLAYbasic as an assessment tool, “Run there and back” and “Hop” were associated with BMI, waist circumference, grip strength, percent body fat, and VO_2peak_ (r ranging from −0.18 to 0.48; all *p* ≤ 0.05; Table 2). The association between “Overhead Throw”, “Kick Ball”, “Balance Walk Backyards” and health indicators ranged from 0.16 to 0.60 (all *p* ≤ 0.05). Using the Passport for Life, “Circuit” and “Run, Stop, Return” were associated with all the health indicators (*r* ranging from −0.17 to 0.55; all *p* ≤ 0.05). “Plank” was associated with all of the health indicators (*r* ranging from −0.24 to 0.44 *p* ≤ 0.05) except for body weight (*r* = −0.17; *p* > 0.05). Of all the FMS tested, only the skill of running was associated with all health indicators. BMI z-score was associated with average PLAYbasic (*r* = −0.18; *p* < 0.01) and average Passport for Life (*r* = −0.27; *p* < 0.01). For both tools, when looking at the average score, 4 out of 6 (67%) health indicators were significantly associated with it. 

However, when adjusting the analysis for age and sex via multiple regressions models, the average score of the PLAYbasic, independent of age and sex, was significantly associated with all of the health indicators including body weight (β −0.30 ± SE 0.4; *p* ≤ 0.05), BMI (β −0.06 ± SE 0.02; *p* ≤ 0.01), BMI z-score (β −2.96 ± SE 1.16; *p* ≤ 0.01), waist circumference (β −0.24 ± SE 0.06; *p* ≤ 0.01), handgrip strength (β −0.33 ± SE 0.11 *p* ≤ 0.01), percent body fat (β −0.15 ± SE 0.04; *p* ≤ 0.01), and VO_2peak_ (β −0.15 ± SE 0.03; *p* ≤ 0.01; Table 3). Similarly, the average score of the Passport for Life was, independent of age and sex, significantly associated with all of the health indicators including body weight (β −10.24 ± SE 3.86; *p* ≤ 0.01; Table 4), BMI (β −2.00 ± SE 0.58; *p* ≤ 0.01), BMI z-score (β −0.20 ± SE 0.06; *p* ≤ 0.01), waist circumference (β −5.91 ± 1.71; *p* ≤ 0.01), handgrip strength (β −8.42 ± SE 2.71; *p* ≤ 0.01), percent body fat (β −4.20 ± SE 1.00; *p* ≤ 0.01), and VO_2peak_ (β 3.10 ± SE 0.72; *p* ≤ 0.01).

Using a stepwise multiple linear regression with the PLAYbasic, “Run there and back” and “Kick Ball” explained 30.4% of the variance of body weight; “Run there and back” and “Kick Ball” explained 26.3% of the variance of waist circumference; average composite score and age explained 29.0% of the variance of handgrip strength; “Run there and back” explained 19.3% of the variance of percent body fat; and finally, “Hop” and sex explained 51.0% of the VO_2peak_. All health indicators were significantly associated with the FMS average *score (p <* 0.05) using the PLAYbasic.

Using a stepwise multiple linear regression with the Passport for Life, age and “Circuit” explained 23.4% of the variance of body weight; “Throw and Catch”, “Run, Stop, Return”, and age explained 26.0% of the variance of waist circumference; “Throw and Catch” and “Run, Stop, Return” explained 17.0% of the variance of handgrip strength; “Run, Stop, Return” explained 16.1% of the variance in percent body fat; and finally, average composite score and sex explained 45.3% of the variance of the VO_2peak_. All health indicators were significantly associated with the FMS average score (*p* < 0.01) using the Passport for Life.

### 3.3. Comparing Associations between Tools and Health Indicators 

Correlations between each health indicator and the average FMS score for both tools were performed. No significant differences were observed when it comes to the association between the average score for each tool and body weight (*p* = 0.42), BMI (*p* = 0.37), waist circumference (*p* = 0.92), handgrip strength (*p* = 0.75), percent body fat (*p* = 0.78), and VO_2peak_ (*p* = 0.99).

**Figure 1:**
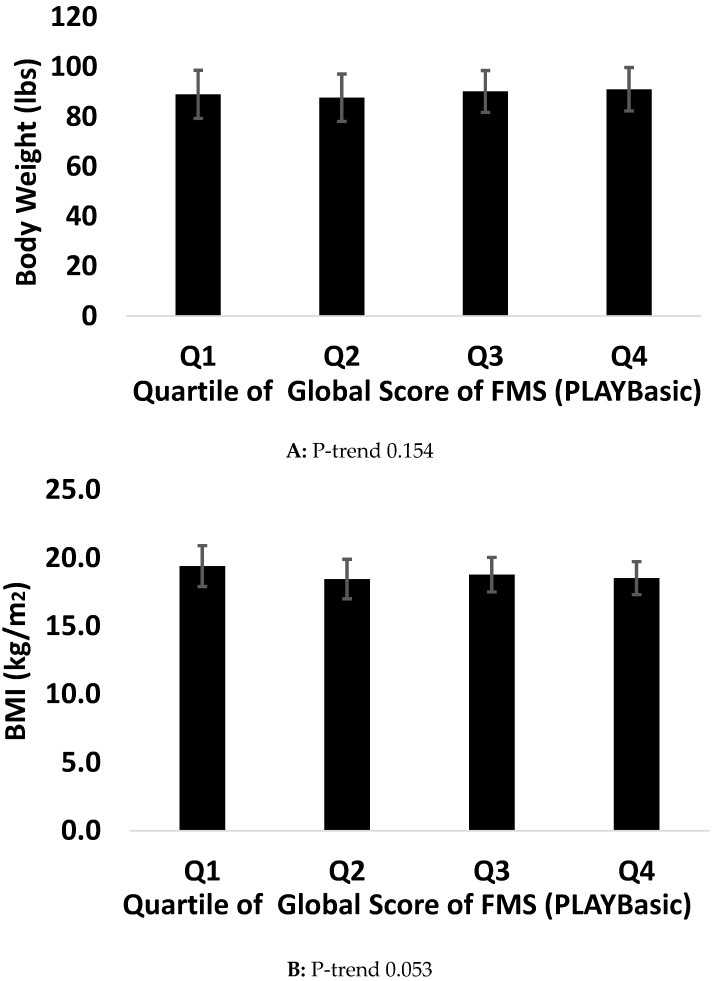

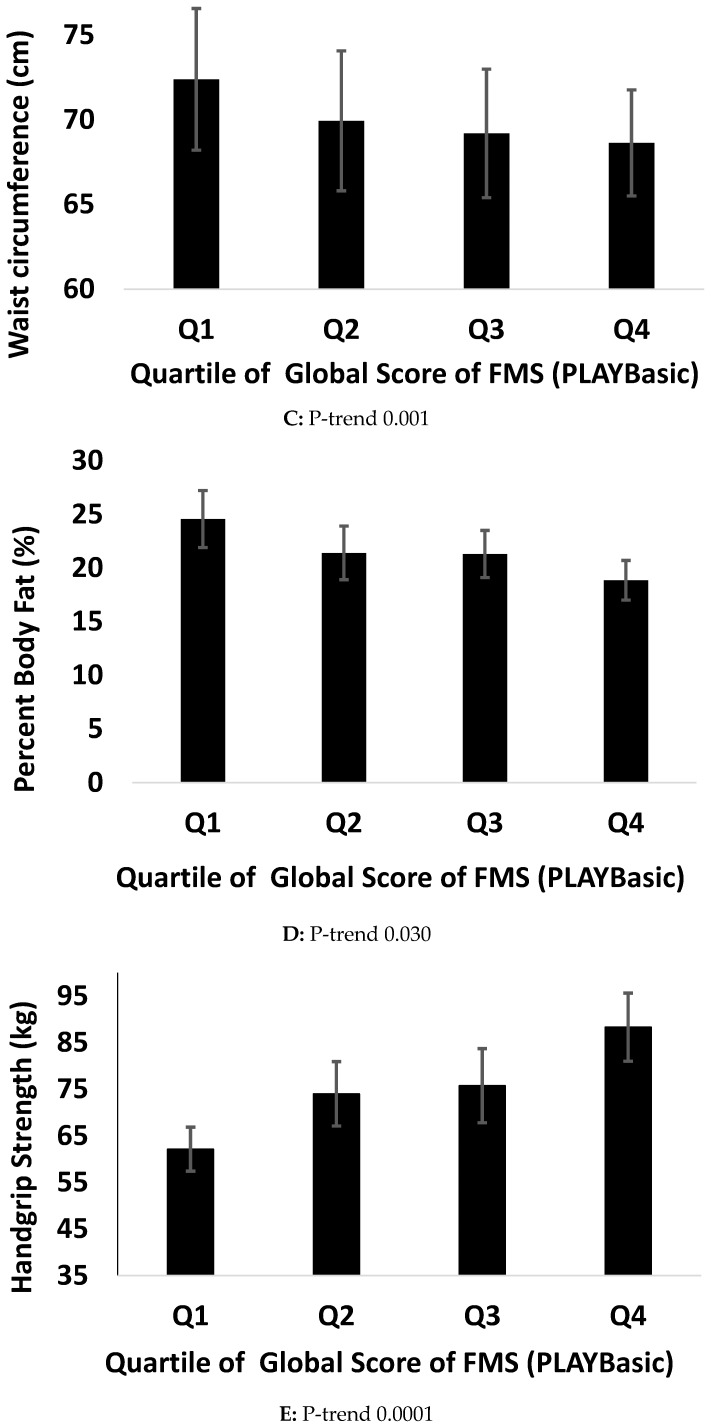

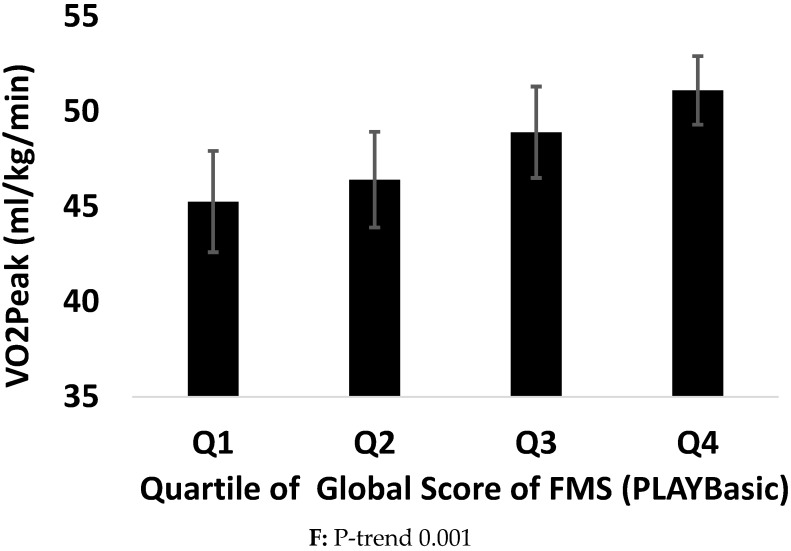
Association between health indicators and FMS using PLAYBasic.

**Figure 2:**
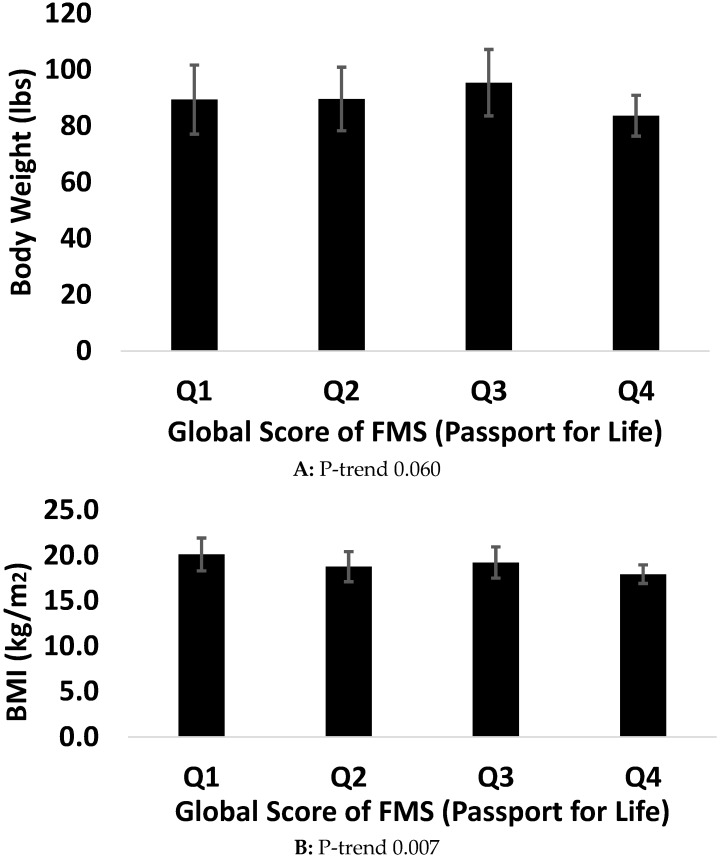

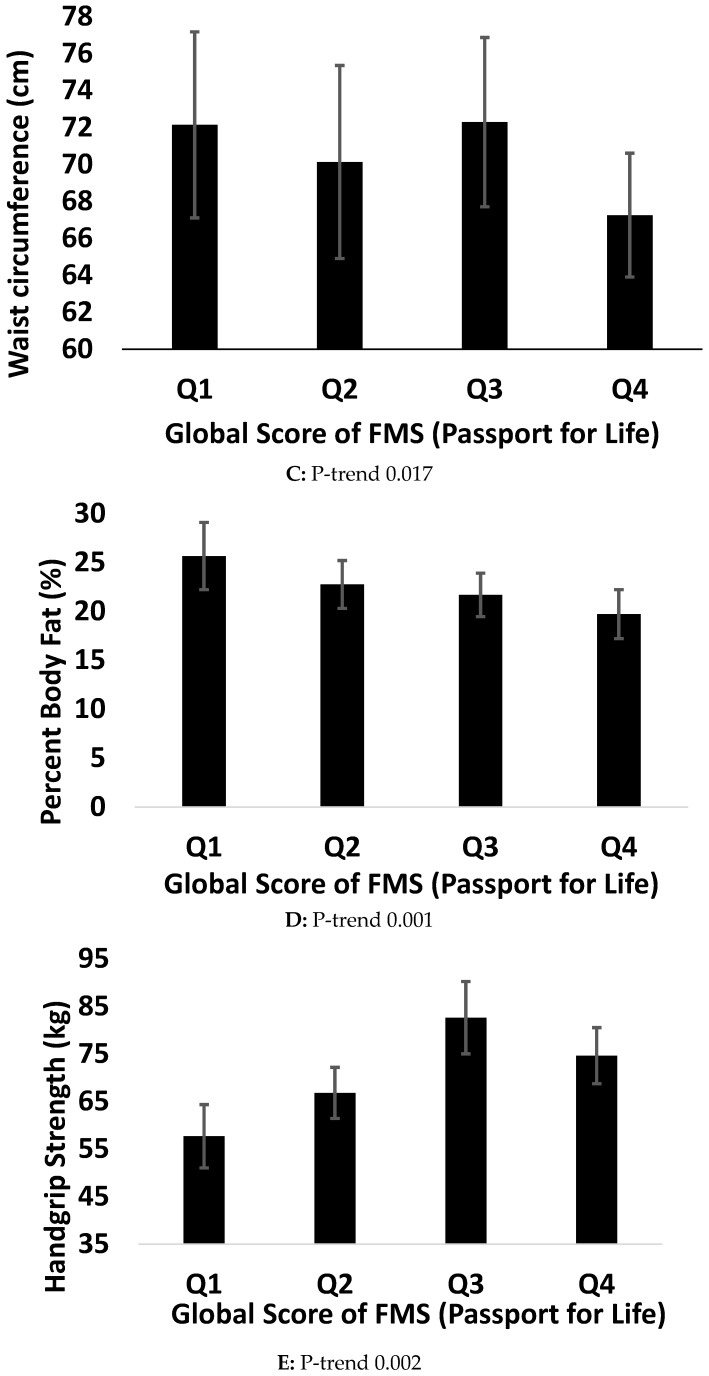

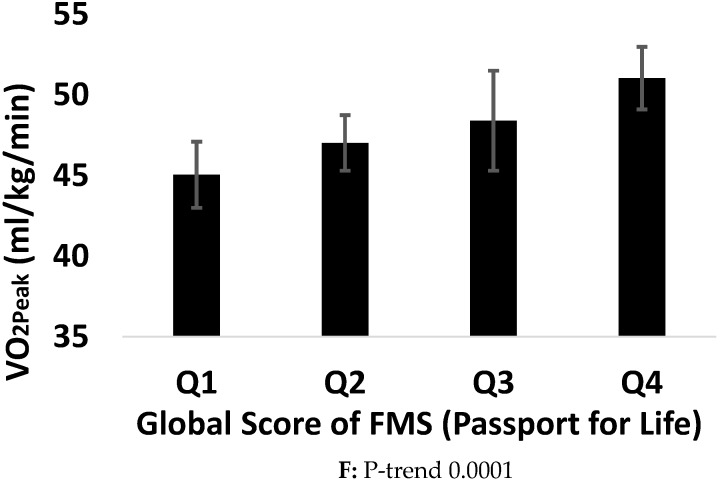
Association between health indicators and FMS using Passport for Life.

Figure 1 and Figure 2 describe P-trend analyses across quartiles of the global score of FMS and each health indicator. A P-trend was observed between the global score of FMS using the PLAYbasic and waist circumference (*p* = 0.001), handgrip strength (*p* = 0.030), and VO_2peak_ (*p* = 0.0001). No such association was observed for body weight (*p* = 0.154) and BMI (*p* = 0.053). Using the Passport for Life, the global score of FMS was associated with BMI (*p* = 0.007), waist circumference (*p* = 0.017), percent body fat (*p* = 0.002), handgrip strength (*p* = 0.001), VO_2peak_ (*p* = 0.001).

## 4. Discussion

The first objective of this study was to investigate the association between FMS in children aged 9 to 12 years old and different health indicators. The results show that the capacity of performing FMS is significantly and independently associated with all health indicators for all ages and sex studied. The second objective of this study was to evaluate whether the correlations between FMS and health indicators were different when using the PLAYbasic or the Passport for Life tools. The results show that the associations between FMS tools and health indicators were not different. Our findings show that FMS are associated with health indicators and suggest that improving FMS could lead to greater health outcomes. These results are of great concern for physical educators and individuals working with a pediatric population who regularly assess FMS in children. 

In Canada and elsewhere, FMS has been popularized in past decades since it was hypothesized that it would be an important factor leading to greater physical activity levels [3,21] which may also positively impact health indicators. In this study, physical activity level was not measured, therefore there is an underlying assumption that the reason why FMS is associated with health markers is because of the association between physical activity level and FMS. However, some controversy exists on the subject. For example, in a systematic review performed by Holfelder et al., (2014), they concluded that a cause-effect relationship between FMS and physical activity is suspected, but has not been demonstrated yet [22]. On the other hand, some have reported that a high level of FMS was associated with more time spent doing physical activities at moderate and vigorous intensities [2]. Discrepancies in the current literature could be explained by the fact that no consensus exists for quantifying FMS. Altogether, these results are of great concern, as it is not clear if there is an association between FMS and physical activity levels, therefore calling for a consensus in how FMS is measured and its implication for health. The right tool to assess FMS is unclear, as there is a lack of research on the validity and reliability of these tools. This study shows that even if the two tools to assess FMS use different tasks, the results are not significantly different. More studies will be needed to compare the association between FMS among other tools, but when it comes to PLAYbasic and Passport for Life, our study shows that one child would not obtain significant difference.

In our study, independent of age and sex, a linear association was found between the global score of FMS and each health indicator using both studied tools, explaining between 9.0% and 49.0% of the variance associated with the health indicators measured. This variance is considerable, given that many other variables such as physical activity level, physical education hours, and participation in organized and non-organized sports could impact the health indicators measured in this study. 

There is a whole body of evidence that studied the association between FMS and body weight and BMI, and most studies suggest a negative association [13,23,24,25]. Although our results confirm these previous data, suggesting a negative association between FMS and body weight and BMI, our study adds to the whole body of evidence by documenting this association with other health indicators and by comparing two fundamental movement skills tools often used in Canada. In our study, we found an independent negative association between FMS and waist circumference and percent body fat. These results are of great concern and reinforce the notion that greater FMS might help prevent the development of some of the main public health concerns in children: Obesity and abdominal obesity. As a result, it is important to encourage in education settings. 

Furthermore, we observed that children with higher FMS display higher muscle strength and cardiorespiratory fitness. Although one may argue that handgrip strength is not representative of overall muscle strength, some data suggest that handgrip strength correlates well with overall muscle strength [26]. These results are of interest, as some data suggests that independent of VO_2peak_, high muscle strength is associated with a lower likelihood of a cluster of health risk factors [27]. Although these results suggest that overall FMS is associated with muscle strength and cardiorespiratory fitness, specific FMS that involve many muscles groups (e.g., hop, circuit) were more likely to be associated with health indicators. 

The current study has some limitations that need to be highlighted. First, the lack of physical activity level of children. In fact, it is possible that some of the associations observed might be mediated through physical activity level and this hypothesis could not be ruled out. Nevertheless, some data support that cardiorespiratory fitness is a stronger predictor of health outcomes compared to physical activity level in children of similar ages. Second, because of the setting in which children were tested, it was challenging to standardize exercise status, food intake, and hydration levels, which might have impacted the associations observed in the current study. Third, the use of bio-impedance to assess body composition is a limitation as it is not the gold standard for this measure. Fourth the small sample size. Fifth, the cross-sectional design does not provide causal relationships. Finally, no biochemical blood indicators were taken in this study. Although this study presents some limitations, it is strengthened by adding unique data to the growing body of research that surrounds FMS. To the best of our knowledge, this is one of the very few studies [16] investigating the comparison of two FMS tools and their associations with health indicators. However, despite the lack of validation of these tools, our data provides relevant insight into the validity between these two Canadian FMS tools. 

## 5. Conclusions

In conclusion, children with a high level of FMS display a better health indicators profile compared to children with poor FMS. Assessing FMS using the PLAYbasic or Passport for Life tool does not impact the association observed with any of the health outcomes measured in this study. More studies are needed to validate these tools and to study whether improvements in FMS lead to improvements in health markers.

## Figures and Tables

**Table 1:** General Characteristics.

*n*	*n* = 145
Age (years)	10.4 ± 1.2
Boys *n* (%)	78 (54.2)
Body weight (kg)	39.8 ± 9.2
Body Mass Index (kg/m^2^)	18.5 ± 2.9
Body Mass Index z-score	0.37 ± 1.0
Waist Circumference (cm)	69.0 ± 8.7
Handgrip Strength (lb)	74.8 ± 21.9
VO_2peak_ (mL/kg/min) ^#^	48.1 ± 3.9
Percent Body Fat (%)	21.5 ± 6.2
PLAYbasic (0–100)	
Run There and Back	62.9 ± 20.6
Hop	61.6 ± 21.6
Overhead Throw	70.2 ± 19.6
Kick Ball	62.3 ± 24.6
Balance Walk Backward	63.0 ± 19.0
Average Score	64.0 ± 15.8
Passport for Life (0–4)	
Lateral Bound	3.4 ± 0.7
Plank	2.6 ± 1.1
Circuit	1.8 ± 1.2
Run, Stop, Return	3.0 ± 0.8
Throw and Catch	3.1 ± 1.1
Kicks	2.7 ± 1.0
Average Score	2.8 ± 0.7

Data are presented as means ± standard deviations for continuous variables and *n* (%) for categorical variables; **^#^** Sample size of cardiorespiratory fitness was: *n* = 42.

**Table 2:** Association between FMS and health indicators.

	Body Weight	BMI	Waist Circumference	Percent Body Fat	Handgrip Strength	VO_2peak_^#^
PLAYbasic						
Run There and Back	−0.18 (0.03)	−0.29 (0.001)	−0.35 (0.001)	−0.45 (0.001)	0.29 (0.001)	0.47 (0.002)
Hop	−0.19 (0.26)	−0.30 (0.001)	−0.32 (0.001)	−0.38 (0.001)	0.24 (0.004)	0.48 (0.001)
Overhand Throw	0.17 (0.044)	0.06 (0.445)	0.021 (0.798)	−0.17 (0.051)	0.33 (0.001)	0.41 (0.008)
Kick Ball	0.21 (0.011)	0.16 (0.054)	0.11 (0.186)	−0.15 (0.071)	0.39 (0.001)	0.49 (0.001)
Balance Walk Backwards	0.01 (0.907)	−0.13 (0.121)	−0.09 (0.295)	−0.26 (0.002)	0.28 (0.001)	0.34 (0.026)
Average Score	−0.002 (0.977)	−0.11 (0.171)	−0.17 (0.037)	−0.33 (0.001)	0.41 (0.001)	0.60 (0.001)
Passport for Life						
Lateral Bound	−0.16 (0.063)	−0.17 (0.042)	−0.21 (0.014)	−0.19 (0.028)	0.15 (0.076)	0.22 (0.164)
Plank	−0.17 (0.43)	−0.24 (0.004)	−0.27 (0.001)	−0.33 (0.001)	0.27 (0.001)	0.44 (0.003)
Circuit	−0.23 (0.007)	−0.29 (0.001)	−0.31 (0.001)	−0.37 (0.001)	0.27 (0.001)	0.54 (0.001)
Run, Stop, Return	−0.17 (0.042)	−0.32 (0.001)	−0.33 (0.001)	−0.47 (0.001)	0.37 (0.001)	0.55 (0.001)
Throw and Catch	0.22 (0.020)	0.043 (0.654)	0.20 (0.036)	−0.15 (0.126)	0.38 (0.001)	0.20 (0.203)
Kicks	0.20 (0.015)	0.12 (0.150)	0.083 (0.322)	−0.19 (0.025)	0.39 (0.001)	0.57 (0.001)
Average Score	−0.09 (0.323)	−0.22 (0.023)	−0.18 (0.520)	−0.37 (0.001)	0.38 (0.001)	0.60 (0.001)

Data are presented as correlations r and (*p*-value); **^#^** Sample size of cardiorespiratory fitness was: *n* = 42.

**Table 3:** Independent associations between FMS and health indicators using PLAYbasic.

	Body Weight	BMI	Waist Circumference	Handgrip Strength	Percent Body Fat	VO_2peak_^#^
Run There and Back	−0.40 ± 0.10 **^‡^**	−0.07 ± 0.01 **^‡^**	−0.24 ± 0.04 **^‡^**	0.16 ± 0.08 *	−0.15 ± 0.03 **^‡^**	0.10 ± 0.03 **^‡^**
Hop	−0.33 ± 0.10 **^‡^**	−0.06 ± 0.01 **^‡^**	−0.20 ± 0.04 **^‡^**	0.13 ± 0.08	−0.12 ± 0.03 **^‡^**	0.10 ± 0.02 **^‡^**
Overhand Throw	0.11 ± 0.12	0.01 ± 0.18	0.26 ± 0.05	0.18 ± 0.09 *	−0.01 ± 0.03	0.60 ± 0.03 *
Kick Ball	0.13 ± 0.10	0.01 ± 0.01	0.01 ± 0.04	0.19 ± 0.07 **^‡^**	−0.01 ± 0.03	0.05 ± 0.03 *
Balance Walk Backwards	−0.25 ± 0.1 *	−0.04 ± 0.02 **^‡^**	−0.15 ± 0.50 **^‡^**	0.17 ± 0.14 *	−0.10 ± 0.30 **^‡^**	0.06 ± 0.03 *
Average Score	−0.30 ± 0.4 *	−0.06 ± 0.02 **^‡^**	−0.24 ± 0.06 **^‡^**	0.33 ± 0.11 **^‡^**	−0.15 ± 0.04 **^‡^**	0.15 ± 0.03

The model was adjusted for age and sex; Data are presented as beta ± SE (p-value) * *p* ≤ 0.05; **^‡^** *p* ≤ 0.01; **^#^** Sample size of cardiorespiratory fitness was: *n* = 42.

**Table 4:** Independent Associations between FMS and health indicators using Passport for Life.

	Body Weight	BMI		Waist Circumference	Handgrip Strength	Percent Body Fat		VO_2peak_^#^
Lateral Bound	−6.51 ± 2.90 *	−1.10 ± 20.50 *	−2.90 ± 1.30 *		2.44 ± 2.26	−1.86 ± 0.82 *	1.22 ± 0.81	
Plank	−6.32 ± 1.80 **^‡^**	−1.02 ± 0.28 **^‡^**	−3.41 ± 0.80 **^‡^**		3.00 ± 1.44 *	−2.02 ± 0.51 **^‡^**	1.41 ± 0.45 **^‡^**	
Circuit	−6.90 ± 1.60 **^‡^**	−1.04 ± 0.25 **^‡^**	−3.46 ± 0.71 **^‡^**		2.90 ± 1.31 *	−196 ± 0.45 **^‡^**	1.65 ± 0.38 **^‡^**	
Run, Stop, Return	−11.30 ± 2.40 **^‡^**	−2.00 ± 0.40 **^‡^**	−6.30 ± 1.05 **^‡^**		5.54 ± 1.97 **^‡^**	−4.22 ± 0.64 **^‡^**	2.91 ± 0.64 **^‡^**	
Throw and Catch	1.32 ± 2.40	−0.05 ± 0.40	0.77 ± 1.10		4.50 ± 1.66 **^‡^**	−0.48 ± 0.66	0.17 ± 0.61	
Kicks	0.64 ± 2.35	−0.04 ± 0.40	−0.80 ± 1.10		4.75 ± 1.81 **^‡^**	−0.84 ± 0.66	1.71 ± 0.60 **^‡^**	
Average Score	−10.24 ± 3.86 **^‡^**	−2.00 ± 0.58 **^‡^**	−5.91 ± 1.71 **^‡^**		8.42 ± 2.71 **^‡^**	−4.20 ± 1.00 **^‡^**	3.10 ± 0.72 **^‡^**	

The model was adjusted for age and sex; Data are presented as beta ± SE (*p*-value) *p ≤ 0.05; **^‡^***p* ≤ 0.01; **^#^** Sample size of cardiorespiratory fitness was: *n* = 42.
